# Evolution in the Management of Patella Fractures

**DOI:** 10.3390/jcm13051426

**Published:** 2024-02-29

**Authors:** Mahak Baid, Sid Narula, Jonathan R. Manara, William Blakeney

**Affiliations:** 1 Aneurin Bevan University Health Board, Wales NP20 2UB, UK; mahak.baid@wales.nhs.uk (M.B.); jonathan.manara@wales.nhs.uk (J.R.M.); 2 Royal Perth Hospital, Perth, WA 6000, Australia

**Keywords:** AO—Arbeitsgemeinschaft für Osteosynthesefragen, TBW—tension band wire, ROM—Range of Motion

## Abstract

Patella fractures usually occur as a result of direct trauma to the anterior knee joint, indirect injury as a result of eccentric muscle contraction, or rapid knee flexion against a contracted quadriceps muscle. The patella functions as part of the extensor mechanism of the knee, where large forces are transmitted, and its subcutaneous nature has made treatment of patella fractures a challenge. In this review article, we evaluate how the management of these fractures has evolved over time and the advantages associated with the various treatment techniques. There are few comparative studies looking at the different treatment types for fractures of the patella, with the goal of achieving a functional extensor mechanism with low rates of post-traumatic arthritis and metal-work irritation.

## 1. Introduction

Patellar fractures constitute 1% of all skeletal fractures. The most common mechanisms are direct trauma to the anterior knee joint or indirect injury resulting from eccentric muscle contraction and rapid knee flexion against a contracted quadriceps muscle [1,2]. Indirect injuries often lead to a transverse fracture or inferior pole avulsion. There is a 2:1 male predominance in patellar fractures [3].

Patients typically present with pain, joint effusion, and a palpable gap in the patella or failure of the extensor mechanism, resulting in an inability to perform a straight leg raise. Patellar fractures exhibit a bimodal age distribution, occurring more frequently in a younger age group with higher energy trauma and a second peak in older age due to osteoporotic fractures [4]. The most common types of multi-fragmentary fractures, namely AO (Arbeitsgemeinschaft für Osteosynthesefragen) type 34-C3 and 34-C1, are associated with high-energy trauma, representing 25% and 23% of all patellar fractures, respectively [4,5,6].

The diagnosis of patellar fractures relies on a combination of clinical findings and imaging. Standard radiographs typically include antero-posterior, lateral, and two oblique knee views. The addition of oblique views enhances sensitivity compared to the standard AP and lateral knee radiographs [7]. The integration of CT scanning assists in classifying the fracture pattern and can impact management plans in as many as 49% of patients with patellar fractures [8].

The subcutaneous nature of the patella and the high level of force transmitted through the extensor mechanism pose challenges in the management of patellar fractures [9]. The extensor mechanism of the knee is a complex structure essential for ambulation. As the largest sesamoid bone in the body, the patella serves multiple functions, including increasing the lever arm of the quadriceps, providing the extensor mechanism with a biomechanical advantage during knee extension, reducing friction between the quadriceps and patellar tendons and the femoral trochlea by acting as a spacer, and offering protection to the tibiofemoral joint against direct trauma [10,11]. The patellofemoral joint experiences substantial compression forces, with three times the individual’s body weight passing through the joint while climbing stairs and over seven times the body weight during squatting [12].

Avulsion fractures of the patella entail the separation of a fragment from the patella, often involving the quadriceps or patellar tendon. Although these fractures are infrequent, they exhibit a higher incidence in adolescents and young adults, particularly among males [13]. Management for these fractures is guided by factors such as the size of the fragment, the degree of displacement, and the patient’s age [14,15]. Conservative treatment, which includes immobilization, protected weight bearing, and physical therapy, is typically employed for smaller, non-displaced fractures. Conversely, larger and displaced fractures necessitate open reduction and internal fixation, where the fragment is reattached using screws, wires, or sutures [16].

Patellar sleeve fractures involve the inferior pole of the patella, characterized by an avulsion of the distal articular cartilage from the under surface and periosteum from the upper surface, along with a complete disruption of the attachment of the patellar tendon. This type of fracture is commonly observed in skeletally immature children [17], constituting 57% of all patellar fractures in children below 16 [18]. Diagnosing the condition poses a challenge, as plain radiographs typically do not depict any signs of a fracture or bony fragment aside from a high-riding patella [19]. Patellar height is classically measured using the Insall–Salvati ratio, which is the length of the patellar tendon divided by the patellar length on the lateral radiographs. The mean ratio in adults is 1.04 (1.01 in males and 1.06 in females). Patella alta is defined by an Insall–Salvati ratio of >1.2, while patella baja is indicated by a ratio of <1.0 [20]. Ultrasound examination of the knee is quick, cheap, easy, and safe, providing a diagnostic tool for these types of injuries [21]. MRI can also serve as a diagnostic modality for these types of injuries [22]. Early identification and treatment are important, as the avulsed fragment leads to bone formation at the distal patella, resulting in the enlargement or duplication of the fragment [23]. These fractures can be managed either conservatively or operatively, depending on various factors such as the size of the osteochondral fragments and the degree of displacement [20]. A prompt diagnosis and appropriate treatment are expected to lead to a full functional recovery.

This review article examines the evidence for the management of patella fractures and how this is evolving with the introduction of modern low-profile, locking plates and compares their biomechanical properties, risks, and benefits.

Periprosthetic patella fractures are rare but catastrophic events following total knee arthroplasty and revision of total knee arthroplasty. These fractures can occur in both resurfaced and un-resurfaced patellae. Patient-, implant-, and surgical technique-related factors contribute to their multifactorial etiopathogenesis. Implant loosening and extensor mechanism disruption are key factors guiding management in these cases. However, they are beyond the scope of this review article.

## 2. Management Options

The primary factors determining the method of treatment include the pre-injury level of patient function, fracture pattern, associated soft-tissue injury, and integrity of the extensor mechanism.

### 2.1. Non-Operative

Undisplaced fractures of the patella, with an intact extensor mechanism (due to an intact retinaculum), can be managed non-operatively [14]. Non-operative management usually involves immobilization in a brace or plaster that restricts flexion for 4 to 6 weeks. The main considerations for non-operative treatment are the degree of fracture displacement and articular congruity. This approach may lead to increased rates of post-traumatic arthritis [24].

It is generally accepted that displaced fractures should be treated operatively. In a study by Pritchell et al., 18 patients with displaced patella fractures were conservatively treated, and all of them developed a 20-degree extensor lag. However, only three patients experienced limitations in their daily activities. This could potentially be attributed to a selection bias for conservative management in low-demand patients [25].

### 2.2. Operative

The origins of patellar fixation can be traced back to 1834 when Barton (1794–1871) first experimented with internal fixation of the patella. However, the patient’s death might be the reason Barton did not publish his procedure [26]. Lister and Trendelenburg performed similar procedures in Germany using drill holes and wire fixation [27]. Numerous techniques of fracture reduction and fixation emerged, but achieving a stable fixation was still a challenge [28,29,30,31]. Different materials were used for fixation and included percutaneous pins, metal loops, kangaroo tendon xenografts, fascial strips, and screws. In 1838, George McClellan (1796–1847) became the first surgeon to successfully perform internal fixation of the patella [32,33].

In 1843, the French surgeon Joseph François Malgaigne (1806–1865) published on percutaneous fixation of a fractured patella using a pair of hooks, later called Malgaigne hooks [34]. The design aimed to realign fragments in cases of displaced patella fracture patterns. Fast forward to 1877, Sir Hector Cameron of Glasgow achieved a milestone by becoming the first to conduct open reduction and internal fixation of a patella fracture using interfragmentary wiring [35]. Richard von Volkmann (1830–1889) described the insertion of a wire suture behind both the quadriceps femoris and the patellar tendons for patellar fracture fixation [36].

The greatest advancement occurred in the 1950s, when Pauwel introduced the anterior tension band technique for patellar fracture fixation [37]. The Arbeitsgemeinschaft fur Osteosynthesefragen/Association for the Study of Internal Fixation (AO/ASIF) subsequently modified and advocated tension band fixation as a rigid construct, enabling early range-of-motion and rehabilitation for patellar fractures [37].

Approximately one-third of patellar fractures require surgical intervention, with joint incongruity and a fracture gap exceeding 3 mm being the most common indications [38]. The goal of fracture fixation is to restore the patient’s anatomy and achieve early mobilization [39,40]. Early mobilization helps prevent stiffness of the knee joint capsule and reduces the cartilage degeneration associated with prolonged immobilization [41].

Following the fracture fixation of the patella, union usually occurs within 8–12 weeks. During this period, the knee is typically taken through around 100,000 cycles of flexion and extension, necessitating a robust fixation method capable of withstanding these high forces [42].

Post operatively patients with patellar fractures have to undergo extensive rehab. There are different variations to the rehab protocol, but the goal is to allow early rehab and the best functional outcome. Our preferred rehab protocol for patients who have undergone surgery include allowing the patients to weight bear as tolerated with knee locked in extension for the first 6 weeks. In the first 2 weeks, they are given a range of motion knee brace locked in extension and knee flexion allowed up to 30 degrees. Between 2 and 6 weeks, the knee flexion is progressively increased by 15 degrees each week with a goal to reach 90 degrees by 6 weeks. At 6 weeks, the knee brace is unlocked while fully weight bearing and completely comes off at 10 weeks. Return to full activities occur at 3–6 months.

### 2.3. Tension Band Wire

**Figure 1:**
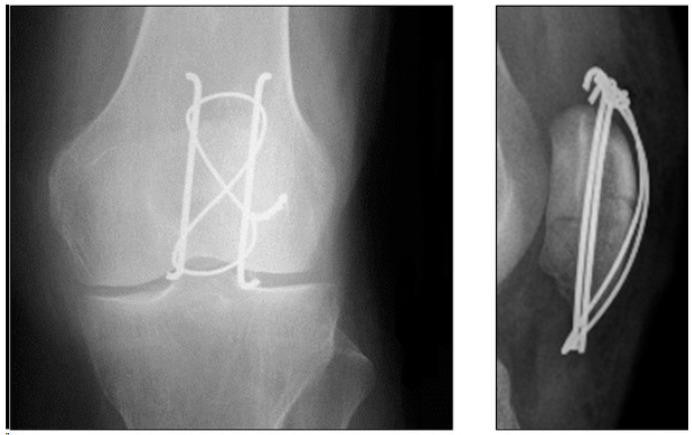
Showcases an example of a transverse patella fracture treated with TBW Fixation. Copyright license-Swiss Medical Weekly supporting association [48].

Transverse patella fractures (AO 34-C1) account for 23% of all patellar fractures. Numerous biomechanical studies have assessed fixation using tension band wire (TBW) in this fracture pattern, and TBW is widely regarded as the gold standard for this ‘simple’ fracture pattern (see Figure 1) [5,43,44,45,46,47].

The biomechanical principle behind TBW is the transformation of tensile forces on the anterior patella surface into compressive forces at the articular surface, achieving dynamic interfragmentary compression [49]. In the traditional TBW (tension band wiring) technique, two K wires are proximally bent in a parallel fashion, and a figure-of-eight cerclage wire is then wrapped around them for stabilization [37]. For comminuted fractures and distal pole fractures, additional circular cerclage or de-tensioning cerclage (McLaughlin) may be used, respectively [50].

The surgical technique when performing TBW is crucial, as demonstrated by Bostrom et al., who emphasized that a wire diameter and tensioning technique are critical factors in the mechanical properties of the construct. They found that a 16-gauge wire was superior to an 18-gauge wire [42].

Complications of TBW fixation include loss of reduction (early fracture displacement has been observed in 22–30% of cases [6,51]), k-wire migration, extensor lag, and soft-tissue irritation. Re-operation rates of up to 65% have been reported [15,52,53]. While the cost of the metalwork is low compared to many orthopaedic implants, the high re-operation rate is associated with the expense of theatre time and exposes patients to the risk of multiple procedures.

### 2.4. Cannulated Screws

**Figure 2:**
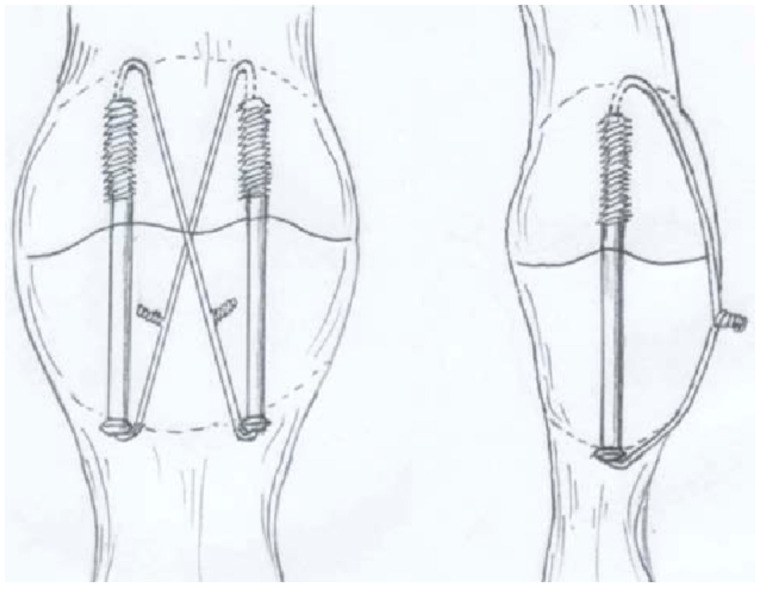
Illustrates the treatment of a transverse patella fracture with cannulated screws and additional wiring. Copyright License-The Korean Fracture Society [58].

Cannulated screw fixation (see Figure 2) was introduced to reduce the soft-tissue irritation caused by k-wires. The theory posits that screws would also provide greater rigidity and improve resistance against tensile loading compared with smooth wires. However, the presence of screw heads has been shown to reduce the construct’s ability to resist gap formation during cyclic loading testing. In this technique, two parallel cannulated lag screws are inserted, crossing the fracture site at 90 degrees to the fracture. The surgeon can then choose to add wires in a figure-of-eight configuration through the screws [54,55,56].

This technique has demonstrated superior stability in biomechanical studies, lower complication rates, and better functional outcomes than TBW alone [38,57].

Dual-pitched buried compression screws with additional suture tension band have demonstrated superior biomechanical behaviours over standard-headed screw fixation, showing increased rigidity of the construct, smaller interfragmentary motion, increased resistance to failure, and greater fixation strength [59]. Martin et al. demonstrated that the mean clinical failure strength for the headless screws construct was almost double that of the headed screws construct [60]. Alayan et al. found comparable results between the two types of fixation, with greater fracture gapping in the buried compression screws and suture fixation compared with wire and cannulated screw constructs [61]. In a retrospective review, Busel et al. evaluated fixation using headed cannulated screws and high-strength nonabsorbable suture, demonstrating high union rates at 96% and a low rate of symptomatic hardware at 8% in a series of 50 patients. Three of the four cases of symptomatic hardware in their study were due to screw prominence. Screw fixation has a similar complication profile to TBW but less metalwork prominence. However, given that screws have a larger diameter compared to wires, there is a higher risk of perforating the articular surface of the patella and causing extension or comminution of the fracture.

**Figure 3:**
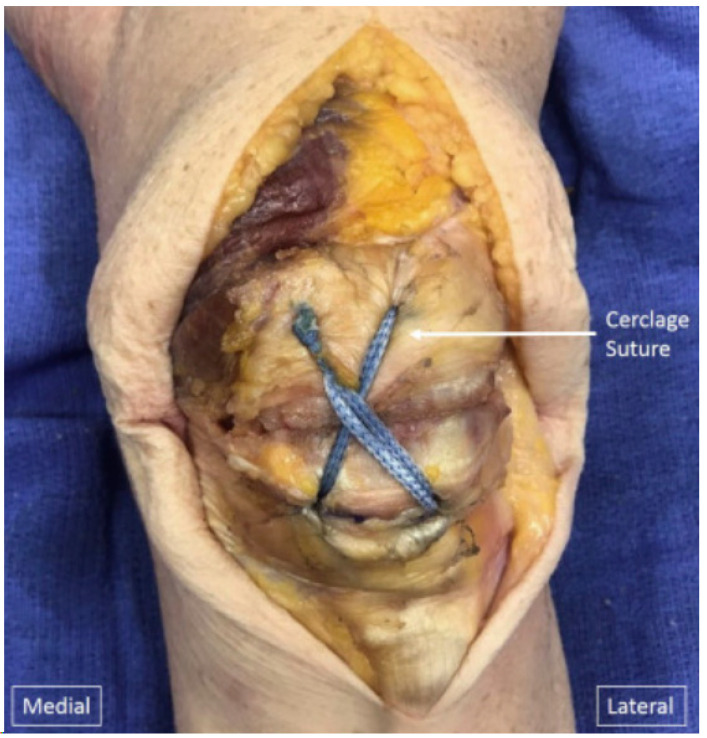
Completed repair of patella fracture fixation. The cerclage suture has been passed through both cannulated screws in a figure-of-eight fashion and tensioned. Copyright by the Arthroscopy Association of North America. Published by Elsevier [66].

Non-absorbable braided sutures, such as FibreTape (Arthrex, Inc., Naples, FL, USA), in isolation have demonstrated comparable strength to TBW, with limited soft-tissue irritation compared to metalwork [62,63,64] (Figure 3). Braided sutures exhibit lesser creep, greater stiffness, and lesser extensibility than standard sutures but have inferior strength to metalwork [65]. Additionally, the radiolucent properties of sutures allow for a more accurate assessment of the fracture reduction radiographically and both intra- and postoperatively compared to metallic hardware. However, when this technique is employed, additional postoperative immobilization is often recommended [61]. This method of fixation may play a role in simple fracture patterns, but may not be appropriate for more complex fractures, either in isolation or in combination with other methods.

### 2.5. Locking Plates

**Figure 4:**
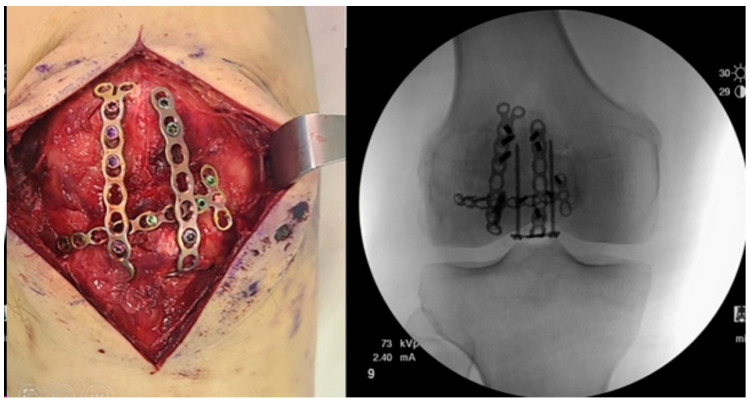
Depicts the fixation of a comminuted patellar fracture using locking compression miniplates and depicts the radiographic image. Copyright BMC Musculoskeletal Disorders [74].

**Figure 5:**
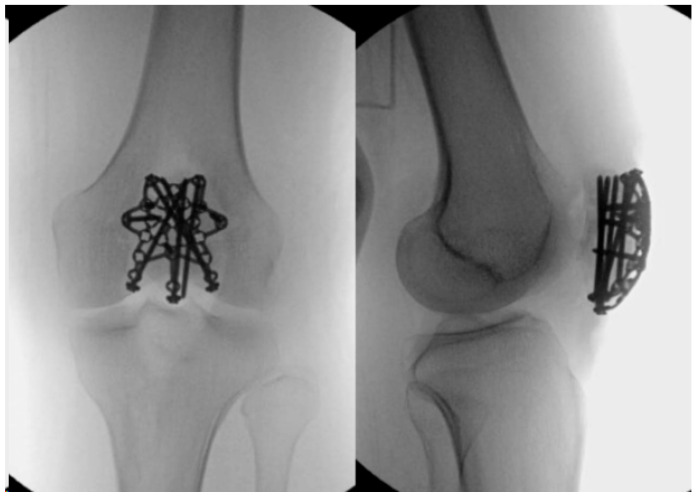
Showcases a low-profile patella plate. Copyright © 2022 Wolters Kluwer Health, Inc. (Waltham, MA, USA) [75].

More recently, variable angle plating systems have been utilized in the management of patella fractures (see Figure 4 and Figure 5). These plates have locking screw holes that allow for up to 15-degree angulation to target screws into small bone fragments [67,68,69,70,71]. The plates can be cut and contoured to address individual fracture patterns and are low-profile to minimize soft-tissue irritation. They enable the insertion of multiple screws (and sutures) into various fracture fragments, making them more likely to be used in the treatment of more complex and multi-fragmentary fracture patterns.

Buschbeck et al. managed 29 complex C3 fractures using anatomically contoured locking plates (Patella SuturePlate™, Arthrex^®^, Naples, FL, USA) over a span of 5 years. All patients underwent follow-up for an average of 19 months (range: 12–48 months). They achieved a mean flexion of 131° (range: 100–150) without any reported reduction loss, mechanical failures, or complications related to the implants. Radiological assessments revealed no signs of post-traumatic osteoarthritis. In seven cases, implants were removed due to subjective soft-tissue irritation [72].

Sumit et al. conducted a review of 20 patients treated with low-profile patellar plates. At the two-year mark, they evaluated the patients radiographically using X-rays and CT scans, along with functional scores. Radiological union, confirmed using CT scans, was observed in all patients at three months. Additionally, two patients experienced superficial surgical site infections at two weeks, and implant impingement was identified in two more patients at three months [73].

Studies have explored patella-specific plates, such as anatomically shaped patellar locking plates, bilateral fixed-angle basket plates, or titanium mesh plates. However, their availability may be limited in various surgical settings.

Yoo et al. have outlined a surgical technique for addressing multifragmentary, comminuted patellar fractures using locking compression miniplates in conjunction with long locking screws/cannulated screws (Figure 4). The study included 20 patients with AO/OTA 34-C3 fractures, who were treated with 2.4 mm LCP Compact Hand miniplate-based internal fixation (DePuy-Synthes^®^, Warsaw, IN, USA) and were followed up for an average of 15 months. These low-profile implants, commonly employed in hand fractures, were utilized alongside other fixation methods such as cannulated screws (nine cases), cerclage (two cases), and wiring (five cases).

The miniplates and locking screws not only provided angular stability but also mitigated the risk of screw pull-out. The number of plates used per case ranged from 2 to 5 (average—2.8 miniplates) based on the fracture patterns. The average time to bone union was 15.6 weeks (range 10 to 40), with no reported cases of fixation failure or postoperative complications, including symptomatic implants. All patients achieved an average range of motion (ROM) of 130 degrees and a mean Lysholm score of 90.4 at the final follow-up. The study suggests that osteosynthesis of patella fractures with Compact Hand miniplates can offer sufficient mechanical stability for early ROM without postoperative complications [74].

### 2.6. Patellectomy

In cases of comminuted fractures or when previous fixation has failed, practitioners may consider a partial or total patellectomy, commonly regarded as a salvage procedure [6]. Total patellectomy is associated with a 50% reduction in quadriceps strength [69]. Additional complications following patellectomy include bowstringing, as well as subluxation or dislocation of the extensor mechanism.

Levack et al. conducted a retrospective review of 64 patellar fractures treated either using internal fixation or patellectomy, with a follow-up ranging from 3.5 to 10.1 years (average 6.2 years) post-surgery. In their study, 45% of the patients achieved a good result, 27% had a fair outcome, and 28% experienced poor results. Overall, patellectomy yielded reasonable outcomes (60% good, 20% fair, 20% poor). Notably, the best results were observed in cases where anatomical reduction of the patellar fracture was achieved with tension band wiring (TBW), underscoring the importance of employing effective surgical techniques [76].

## 3. Comparison of Techniques

### 3.1. Biomechanical Studies

Ivan et al. conducted a comparative analysis of various techniques for patella fracture fixation using twelve human cadavers with simulated transverse patellar fractures [77]. The cadavers underwent treatment with either tension band wiring (TBW) or cannulated screw fixation. The study revealed that the mean anterior and posterior interfragmentary pressure differences between the two groups were non-significant. However, the group treated with cannulated screws exhibited significantly less displacement at the fracture site at flexion angles of 45°, 60°, and 75°. The conclusion drawn was that screws caused less posterior fracture site displacement at specific flexion angles and provided additional compressive strength when compared to TBW [56,78].

Banks et al. conducted a comparison between open reduction and internal fixation of the patella using a locking plate and fixation with cannulated screws and tension band wiring (TBW). While the load at clinical failure did not show a significant difference between the two groups, the ultimate strength of fixation was significantly higher in the plate fixation group [79].

Wagner et al. compared locking plates and cannulated screws with tension band wiring (TBW) through the screws. The hybrid group, consisting of a cannulated lag screw and TBW, showed statistically significant higher displacement than the locking plate group after 100 cycles [5,80]. During cyclic loading, five out of the eight hybrid constructs failed, while all constructs in the plate osteosynthesis group withstood the test fixation stress. The conclusion drawn was that a patella locking plate is a safe and effective treatment for patella fractures [81,82].

### 3.2. Clinical Studies

There are few trials directly comparing the different surgical techniques, and to the authors’ knowledge no randomised control trials.

Meng et al. conducted a retrospective comparison study involving a total of 87 cases of 34-C2 and 34-C3 comminuted patellar fractures to evaluate the effects of two different treatments: a titanium cable tension band with cerclage method and an interfragmentary screws with X-shaped plating technique. They included patients with closed fractures, <21 days with an intra-articular step >2 mm or a gap of >3 mm, and no history of previous injury or surgery in the knee joint. At the 2-year follow-up, the study found no significant difference between the two groups in terms of Lysholm knee score, range of motion (ROM), and complications, including implant-related complications, osteoarthritis, and knee contraction [83].

Meng et al. conducted a comparative study assessing complications and the effectiveness of three different methods used to treat transverse patellar fractures in 108 patients. The methods included titanium cable tension bands (group A), compression screws with titanium cable cerclage (group B), and X-shaped plating technique (group C). No significant difference was noted in terms of postoperative articular step-off, Lysholm score, and range of motion at 24 months among all groups. At the final follow-up, the study reported 12 (31.6%) symptomatic implant complications in group A, 6 (16.7%) in group B, and 2 (5.9%) in group C. All three methods were effective in achieving rigid fixation and enabling early functional rehabilitation. However, the X-plate technique demonstrated the lowest risk of symptomatic implant complications and was considered a safe and effective alternative for the internal fixation of transverse patellar fractures [84].

Lorich et al. conducted a prospective cohort study comparing a novel patella fixation technique using a plate construct that spans half of the patella surface with traditional tension band wiring. The plate group showed superior Knee Outcome Survey Activities of Daily Living Scale (KOS-ADLS) scores throughout the study, along with significant improvements in functional testing and reduced anterior knee pain. The novel construct involves a low-profile mesh plate, providing multiplanar and interfragmentary fixation, addressing inferior pole comminution, and minimizing disruption to patellar vascularity. At 12 months, thigh circumference was significantly higher, and anterior knee pain was significantly lower in the plate group. The study concludes that the novel fixation construct improves patient outcomes postoperatively, offering enhanced stability, reduced soft-tissue irritation, and minimized disruption to patellar vascularity, leading to decreased anterior knee pain [85].

Balgovind et al. conducted a systematic review focusing on the clinical outcomes of patients treated with plate osteosynthesis for patellar fractures, with a minimum follow-up of three months. The review included 20 studies, comprising 7 prospective and 13 retrospective articles, involving 533 patients with 534 patellar fractures treated using plate osteosynthesis. The majority of fractures treated were of AO-type 34C. The findings of the systematic review indicate that plate osteosynthesis demonstrated superior clinical outcomes, lower complication rates, and higher union rates when compared to tension band wiring for the management of patellar fractures [86].

Tsotsolis et al. conducted a systematic review on the functional outcomes and complications of plate fixation in patellar fractures, adhering to the PRISMA guidelines and searching databases such as MEDLINE, EMCare, CINAHL, AMED, and HMIC. The review revealed that the plating of patellar fractures is linked to satisfactory range of movement (ROM), favourable postoperative function, and low pain levels. The study identified a 10.44% complication rate and a low reoperation rate, with reoperations mainly performed for metalwork removal. The findings suggest that open reduction and internal fixation (ORIF) with plating for patellar fractures is a safe alternative, associated with a lower complication and reoperation rate compared to tension band wiring (TBW). The authors emphasize the need for future randomized prospective studies to validate the results of this systematic review [87].

### 3.3. Sequelae of Patella Fracture

Patients who have experienced patellar fractures often exhibit a high occurrence of postoperative patellofemoral joint osteoarthritis (OA) as a sequelae, with reported residual pain in thirty to fifty percent of cases [78,88].

Larsen et al. conducted a comprehensive study involving 6096 patellar fractures, with a mean follow-up of 14.3 years. In their case-matched series, they observed a significantly higher incidence of osteoarthritis among those who had suffered a patellar fracture, with an 83% increased rate of total knee replacement, particularly within the first 5 years. These patients also demonstrated a four times increased incidence of knee arthroscopy compared to the control group and exhibited decreased knee functional scores [5].

Non-union or delayed union in closed, operatively treated patella fractures is rare, with one series reporting a rate of less than 1% [67]. Torchia and Lewallen observed a notable correlation with open fractures. In their series, 2 out of 28 patients (7%) who underwent open reduction and internal fixation (ORIF) for open patellar fractures experienced non-union [89]. In cases of delayed union, immobilization for a certain period may be considered to facilitate fracture union. If this approach proves ineffective, a subsequent attempt at fixation can be undertaken, often yielding favourable outcomes [90].

The incidence of infection after fixation of patella fractures ranges from 2 to 10% in the literature [11,53,91]. Superficial infections are often successfully treated with local wound care and oral antibiotics, whereas deep infections require operative debridement and a period of intravenous antibiotics with retention of hardware until fracture union.

Avascular necrosis of the patella is a rare diagnosis. It has been described as a complication of trauma [92], post-total knee replacement, and as an idiopathic disease [93,94], and treatment is mostly observation as they revascularize for 2 years.

## 4. Conclusions

Patella fractures present with a spectrum of severity. They are seen in a variety of age groups and in patients with differing demands and they remain a challenge to treat. There are multiple treatment options and an individualised approach must be taken. It remains to be seen if patella plating will justify the additional costs compared to traditional techniques in the long term. Comparison between techniques remains challenging with a variety of treatment methods and rehabilitation protocols being used in differing population groups and fracture patterns. There are few comparative studies and, therefore, evidence of the superiority of different techniques is lacking. The goal of rigid fixation whilst minimising metalwork irritation and subsequent reduced return-to-theatre rates should prove cost effective in the long term.

## Data Availability

No new data were created or analyzed in this study. Data sharing is not applicable to this article.
